# Amphiphilic Janus nanoparticles for a superhydrophobic coating on the enamel surface to prevent caries

**DOI:** 10.1016/j.mtbio.2025.101627

**Published:** 2025-03-06

**Authors:** Chenhui Chen, Yuqi Xie, Qinfeiyang Zhang, Junyan Cao, Jixing Lin, Xian Tong, Yihao Wu, Li Zhu, Peng Gao, Jianfeng Ma

**Affiliations:** Institute of Stomatology, School and Hospital of Stomatology, Wenzhou Medical University, Wenzhou, 325027, China

**Keywords:** dental caries, Caries prevention, Superhydrophobic materials, Janus amphiphilic nanoparticles, Enamel binding peptide

## Abstract

Bacteria are pivotal in the etiology of dental caries, underscoring the critical importance of effective plaque control in mitigating dental tissue diseases. Superhydrophobic materials, with their exceptional properties of non-wettability, antibacterial activity, and self-cleaning capabilities, present a promising approach for caries prevention. However, their clinical application remains constrained by challenges in achieving stable adhesion to tooth surfaces. Inspired by the natural adhesion mechanisms of acquired salivary pellicle (ASP), we synthesized Janus amphiphilic superhydrophobic nanoparticles comprising 1H,1H,2H,2H-perfluorooctyltriethoxysilane (FOTS), nano-silica, and the enamel binding peptide DDDEEKC-Peg(2000)-COOH. These nanoparticles were engineered to form a superhydrophobic film (FOTS-SiO_2_-Peptide) on tooth surfaces. Experimental analyses revealed rapid adsorption of these nanoparticles onto enamel, with their exposed surfaces creating a durable superhydrophobic layer that remained stable under diverse thermal conditions. Topical application of FOTS-SiO_2_-Peptide significantly reduced the incidence and severity of dental caries while preserving oral microbiota diversity and avoiding adverse effects on surrounding mucosal tissues. Safety and efficacy evaluations confirmed the biocompatibility of these Janus nanoparticles, highlighting their potential as a novel biomedical solution for caries prevention.

## Graphical abstract

**Figure:**
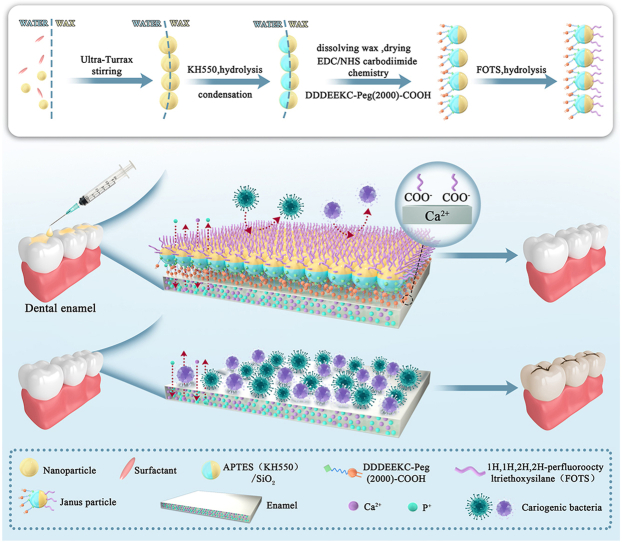

## 1 Introduction

Dental caries is a multifactorial and dynamic disease driven by bacterial biofilms on tooth surfaces [1]. Cariogenic bacteria, as primary contributors [2], metabolize dietary carbohydrates into acids [[2], [3], [4]], disrupting the local microecological balance and initiating enamel demineralization [1,5,6]. Dental caries ensue if the equilibrium between demineralization and remineralization is not restored [5,7]. During biofilm formation, Streptococcus species are the first to colonize [8], marking the transition from initial plaque to mature cariogenic biofilm [2,3,9].

The broad-spectrum antibacterial agent chlorhexidine can cause tooth staining and taste disturbances [10,11], while prolonged use of triclosan may disrupt the balance of oral microbiota [11,12]. Similarly, fluoride effectively reduces caries incidence [13,14] but is associated with the selective growth of fluoride-resistant strains and adverse effects such as dental and systemic fluorosis [15,16]. Although advanced materials with enhanced antibacterial properties and remineralization capabilities have been developed, these materials primarily function as fillings in teeth that have already experienced caries [[17], [18], [19]].

To address these challenges, novel antimicrobial approaches are required to prevent biofilm formation and bacterial adhesion while preserving the ecological balance of oral microbiota. Due to their low surface energy properties, superhydrophobic materials offer a promising alternative. This non-lethal antimicrobial approach presents unique clinical advantages: it reduces the initial adhesion and colonization of microorganisms on material surfaces and serves as a preventive measure to inhibit the occurrence of early dental caries [[20], [21], [22]]. Inspired by natural adhesion mechanisms, the salivary-acquired pellicle (SAP) can tightly cover the tooth surface. Among its components, Statherin, a key salivary protein, is a fundamental constituent of SAP. The first six amino acids of this protein (DDDEEKC) adopt an α-helix conformation and possess the ability to recognize hydroxyapatite (HA). Research has demonstrated that this sequence exhibits strong adsorption and fixation properties on the surface of hydroxyapatite [23,24], offering a foundation for constructing superhydrophobic coatings.

Janus amphiphilic nanoparticles combine dual surface properties [25], with a hydrophobic side to form a superhydrophobic interface and a hydrophilic side to enhance adhesion to enamel. These features make them ideal for developing stable superhydrophobic films. Modifying silica nanoparticles with peptide DDDEEKC and trifluorooctyl triethoxysilane synthesized stable and functional Janus nanoparticles. Peptides were stabilized using Peg(2000)-COOH, providing potential sites for reaction with amino groups. *In vitro* and *in vivo* results demonstrated that Janus nanoparticles significantly inhibited Streptococcus adhesion and biofilm formation on enamel surfaces. Biosafety evaluations confirmed their excellent biocompatibility, including CCK-8 assays and blood compatibility tests. In the rat model, topical application of Janus nanoparticles effectively reduced caries severity without adverse effects. This safe and efficient strategy offers a clinically viable solution for caries prevention through a simple dip-coating method.

## 2 Materials and methods

### 2.1 Materials

Hanks' Balanced Salt Solution, 3-Aminopropyltriethoxysilane, 1-Ethyl-(3-dimethylaminopropyl) carbodiimide Hydrochloride, ethylene glycol, citric acid, and glacial acetic acid were obtained from Shanghai Macklin Biochemical Technology Co., Ltd. Didodecyldimethylammonium bromide and paraffin were procured from Shanghai Aladdin Biochemical Technology Co., Ltd. Acid Orange II was supplied by Cato Research Chemicals Inc. Trifluorooctyltriethoxysilane and N-Hydroxysuccinimide were sourced from Shanghai Yuanye Biotechnology Co., Ltd. Artificial saliva (pH 6.8) was purchased from Beijing Leagene Biotechnology Co., Ltd. DDDEEKC-Peg(2000)-COOH and SH-Peg(2000)-COOH was obtained from Nanjing Genscript Biotechnology Co., Ltd. Additional materials included a CCK-8 detection kit (from a specified supplier), Brain Heart Infusion (BHI) broth (from a selected supplier), Penicillin-Streptomycin Solution (100 × , double antibiotic) from Wuhan Procell Biotechnology Co., Ltd., China and MSA medium from Qingdao High-tech Industrial Park Haibo Biotechnology Co., Ltd., China.

### 2.2 Preparation of paraffin pickering emulsion droplets

SiO_2_ nanoparticles of specific sizes were synthesized following the protocol described by Stöber et al. [26] and Garcia et al. [27]. Paraffin Pickering emulsions were prepared based on Granick's method [28,29], using paraffin and SiO_2_ nanoparticles as raw materials, with CTAB as the surfactant. Weakly attached particles were removed through suction filtration and washed with ultrapure water. This process was repeated three times. The resulting emulsion was dried at a low temperature and stored in the dark for subsequent use.

### 2.3 Preparation of NH_2_-functionalized SiO_2_

**Fig. 1:**
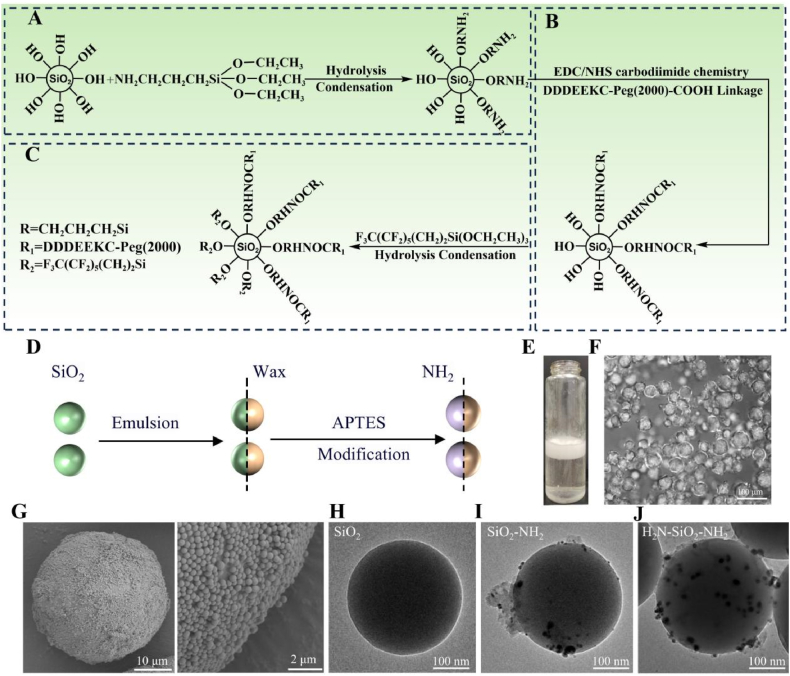
(A) Reaction mechanism of KH550-modified SiO_2_ NPs, (B) Carbon Diimide Chemistry, and (C) FOTS-modified SiO_2_ NPs. (D) Synthesis schematic of Janus silica nanoparticles on the Pickering emulsion interface. (E) The appearance of o/w emulsions stabilized by nano-silica particles and surfactant DDAC. (F) Optical microscope images of emulsion. (G) SEM images of emulsion. (H) TEM images of SiO_2_ nanoparticles. (I, J) TEM image of silver nanoparticle labeled nanoparticles.

To prepare NH_2_-functionalized SiO_2_, KH550 (4 wt%) was added to 100 mL ethanol (90 % v/v), and the pH was adjusted to 3–3.5 using acetic acid [30,31]. After standing for 6 h, 1 g of paraffin Pickering emulsion was added at room temperature and stirred at 500 rpm for 10 h. The reacted droplets were centrifuged to collect SiO_2_ particles detached from paraffin. Ethanol was used to remove acetic acid, and residual paraffin was cleared using tetrahydrofuran. The resulting SiO_2_ particles were functionalized with NH_2_ groups on one side. The reaction mechanism is shown in Fig. 1A.

To quantify NH_2_ groups, 10 mg of NH_2_-functionalized SiO_2_ was reacted with Acid Orange solution (1 mg/mL, pH 4.5–5.5) on a shaker for 4 h at room temperature. The mixture was washed with hydrochloric acid and centrifuged until the supernatant was clear. The SiO_2_ particles were then treated with 0.4 mg/mL NaOH solution (pH 11) until the supernatant was transparent. The optical density (OD) of the supernatant was measured at 485 nm using a spectrometer. A calibration curve was constructed with Acid Orange standard solutions (0.2–0.8 mM). Each sample was measured in triplicate, and the NH_2_ content was calculated from the OD_485_ values.

According to the aforementioned method, KH550 was first subjected to hydrolysis treatment, followed by the addition of 0.5 g SiO_2_ particles, thereby preparing KH550-functionalized SiO_2_ particles.

### 2.4 Preparation of peptide-functionalized SiO_2_

Peptide-functionalized SiO_2_ particles were synthesized *via* carbodiimide coupling [32,33]. The reaction was performed with a molar ratio of NH_2_: COOH = 1:3, EDC: COOH = 6:1, and EDC: NHS = 1:1. The mixture was incubated at 4 °C for 21 h with continuous shaking. The resulting peptide-functionalized SiO_2_ particles were dried and stored in the dark. The reaction mechanism is illustrated in Fig. 1B.

According to the aforementioned method, 5 mg of the particles above were separately weighed and reacted with SH-Peg(2000)-COOH. The reaction product was then formulated into a solution with a concentration of 1 mg/mL. Subsequently, gold nanoparticles modified with citric acid were added at a volume ratio of 1:10, mixed, and reacted for 24 h, ultimately acquiring gold nanoparticle-labeled silica [34].

### 2.5 Synthesis of amphiphilic SiO_2_ janus particles

Peptide-functionalized SiO_2_ particles were dispersed in 100 mL of a 50 % (v/v) aqueous ethylene glycol solution and sonicated for 30 min. PFOTS (4 wt% relative to SiO_2_) was added to the dispersion, and the pH was adjusted to 3–3.5 using acetic acid. The mixture was stirred at 500 rpm overnight at room temperature. The resulting superhydrophobic Janus amphiphilic particles were collected, and the reaction mechanism is depicted in Fig. 1C.

### 2.6 Characterization

The surface morphology and elemental composition of the samples were analyzed using scanning electron microscopy (SEM, Nova Nano SEM450, USA) and transmission electron microscopy (TEM, FEI Talos F200s, USA) equipped with energy-dispersive X-ray spectroscopy (EDS). Surface composition and valence states of elements were assessed by X-ray photoelectron spectroscopy (XPS, Escalab 250Xi, USA). The crystal structure was examined using an X-ray diffractometer (XRD, Rigaku SmartLab SE, Japan). Zeta potential and nanoparticle size were measured with a Malvern particle size analyzer (Nano ZS90, UK). Water contact angles were determined using a contact angle meter (SDC-200 S, Sindin, China).

### 2.7 Preparation of bovine tooth slices

Anterior incisors from mature cattle were rinsed and sectioned into uniform blocks along the labial surface. The blocks were sequentially polished with sandpaper of varying mesh sizes to produce 5 mm × 5 mm bovine tooth slices with a thickness of 1 mm.

### 2.8 Dip coating

With the enamel surface facing upward, the bovine tooth slices were immersed in 10 mg/mL of SiO_2_, FOTS-FOTS, SiO_2_-Pep, and Janus particle solutions for 10 min, respectively. Subsequently, the slices were rinsed with deionized water, dried, and prepared for subsequent analysis.

### 2.9 Hydrophobicity test

Water contact angles on the surfaces of different bovine tooth slices were measured. The roll-off angles of water droplets on the T-Pep/FOTS coating were recorded.

### 2.10 Adsorption property test

Enamel slices (6 per group) were soaked in 10 mg/mL of SiO_2_, FOTS-FOTS, SiO_2_-Pep, and Janus particle solutions for 10 min, respectively. Three slices from each group were analyzed directly without rinsing to assess attachment and hydrophobic properties *via* SEM. The remaining slices were rinsed with PBS thrice and analyzed using SEM, EDS, XPS, FTIR, and hydrophobicity tests.

### *2.11* *In vitro* mineralization performance study

Enamel slices were ultrasonically cleaned, and their working surfaces were etched with 37 wt% phosphoric acid for 40 s, rinsed, and dried to produce a demineralized enamel model. Experimental groups included (1) the control group, (2) the T-Pep group, and (3) the T-Janus group, with six replicates per group. Coated enamel samples were placed in a 24-well plate with 2 mL of artificial saliva added to each well. The plates were incubated at 37 °C, with the saliva refreshed every 8 h. After 3 days of mineralization, the enamel slices were rinsed, dried, and analyzed. Three slices from each group were subjected to XRD analysis (30 kV, 40 mA, 2θ = 10–80°, scan speed 5°/min), and the results were processed using MDI Jade 6.5 software. Surface morphology was observed *via* SEM. Three enamel slices per group were treated with 6 wt% citric acid for 1 min to assess acid resistance, followed by SEM observation. Calcium and phosphorus concentrations in the acid-treated enamel were quantified using inductively coupled plasma optical emission spectrometry (ICP-OES, Agilent 5800, USA).

### *2.12* *In vitro* evaluation of cytotoxicity

Extracts of T, T-NH_2_-Pep, and T-Janus were prepared in advance. L929 cells were seeded at a density of 10,000 cells per well in 96-well plates and incubated for 24 h. The medium was then aspirated, and the cells were rinsed with phosphate-buffered saline (PBS). Subsequently, 100 μL of each extract was added to the wells and incubated for another 24 h in a CO_2_ incubator. Cell viability was assessed using the CCK-8 assay, with absorbance measured at 450 nm (OD_450_). The morphology and integrity of the adherent cells were observed under an inverted fluorescence microscope.

### 2.13 Evaluation of hemolysis

Whole blood was collected from SD rats *via* venous sampling and centrifuged to isolate red blood cells. Positive and negative controls were prepared by mixing 20 μL of blood cells with 1 mL of ultrapure water and normal saline, respectively. Experimental groups were prepared by mixing 20 μL of blood cells with Janus particle suspensions at concentrations of 50 μg/mL, 75 μg/mL, and 100 μg/mL. The mixtures were incubated at 37 °C for 4 h, and the absorbance of the supernatant was measured at 542 nm. The hemolysis rate (%) was calculated using the formula:

$$Hemolysis\;rate\left(\%\right)=\frac{\left({OD}_{0}-{OD}_{1}\right)}{\left({OD}_{2}-{OD}_{1}\right)}\times 100\%$$

where OD_0_ is the absorbance of the experimental group, OD_1_ is the negative control, and OD_2_ is the positive control.

### 2.14 Inhibition of single and mixed bacterial biofilms

Single bacterial biofilm inhibition: *S. mutans* UA159 was cultured in BHI medium and resuspended in BHIS medium to adjust the OD600 value to 0.5. The bacterial suspension was diluted with BHIS medium at a 1:100 vol ratio. Sterilized bovine enamel slices with the working surface facing up were placed in 48-well plates, with 1 mL of BHIS bacterial suspension added to each well. Experimental groups included a blank control group, a T-Pep-SiO_2_ group, and a T-Pep/FOTS group, each with six parallel enamel slices. All groups were incubated in an anaerobic environment at 37 °C for 8 h.

Two slices per group were subjected to live/dead bacterial staining to observe bacterial distribution, with images captured *via* SEM after fixation and dehydration. The remaining slices were sonicated in 5 mL PBS for 60 s to disperse bacteria from the biofilm. Serial dilutions were plated and incubated in a CO_2_ incubator for 48 h to quantify bacterial colonies.

Mixed bacterial biofilm inhibition: Continue to resuscitate *S. sobrinus* BNCC353916 and *S. sanguinis* ATCC19556. Mix the aforementioned three bacteria in equal proportions and adjust to an OD_600_ value of 0.5. The subsequent steps are the same as those described for single bacterial biofilm inhibition.

### *2.15* *In vivo* anti-caries and stability effect

Animal experiments were conducted following ethical approval (approval number: WYDW2024-0333). Twelve specific pathogen-free (SPF) rats, aged 6–8 weeks and weighing approximately 160 g, were obtained from SPF (Beijing) Biotechnology Co., Ltd., China. For three consecutive days, rats were given drinking water and chow containing penicillin-streptomycin solution (100 × double antibiotic) to remove endogenous oral bacteria. Subsequently, a multi-species bacterial suspension (100 μL, 1 × 10^9^ CFU/mL for each species) was inoculated onto each mouse's teeth three times daily (morning, noon, evening) for 3 days. Rats were fed a normal diet and sterilized water during this period. All rats were fed a cariogenic diet (D160323, Dyets Biotechnology (Wu Xi) Ltd., China) and 5 % sucrose water starting from the inoculation of bacteria until the end of the experiment.

The experimental groups included the control group, tooth brushing group, tooth brushing + Janus particles group, and tooth brushing + sodium fluoride group (2 % sodium fluoride solution). Rat in groups (2), (3), and (4) underwent routine oral cleaning after anesthesia with 10 % chloral hydrate (0.4 mL/100 g body weight, intraperitoneal injection). According to the method described by Amano et al. [35], an electric toothbrush (Baier, A9, China) was used, with most of its bristles trimmed to produce a working surface suitable for the rat anatomy. Sodium fluoride was applied to the teeth in group (4), while Janus particle solution was injected into the teeth in group (3) for 5 min. Treatments were administered daily from days 7–9, then every two days until the experiment ended on 24 days. Rats were fasted for 30 min before and after treatments. Body weights were recorded on 1, 4, 7, 9, 15, 21, and 24 days.

Dental plaque samples were collected, and bacterial colony counts were performed on 3, 6, 9, 15, 21, and 24 days, following previously described protocols [36,37]. At the end of the experiment, Rats were sacrificed by isoflurane overdose inhalation. Maxillary teeth, jawbones, oral mucosa (palate, tongue, cheek), and major organs (heart, liver, spleen, lungs, kidneys) were harvested [38,39]. The extent of damage to dental caries was scored using Keyes’ scoring method. The biological safety of the treatments was evaluated through hematoxylin-eosin (H&E) staining of oral mucosa and major organs.

To investigate the stability of the enamel surface coating in the actual oral environment further, we conducted the following experiments: rats were anesthetized with isoflurane, and then Janus nanoparticles were coated on the surface of the rat teeth. After the rats regained consciousness, the timing began. Rats with teeth surfaces loaded with superhydrophobic coatings were euthanized at different time points (0 h, 2 h, and 4 h), and then their maxillary molars were extracted. The hydrophobic angles of the tooth surfaces were tested.

### 2.16 Statistical analysis

Data were expressed as mean ± standard error. Statistical analyses were conducted using SPSS software (IBM Corp., Armonk, NY, USA). The Shapiro-Wilk test was first used to assess data normality. One-way analysis of variance (ANOVA) with post hoc multiple comparisons (Tukey HSD) was applied to detect group differences. For colony-forming unit (CFU) counts, data were log-transformed prior to analysis. Graphs were generated using Origin 2021 software. A significance level of p < 0.05 was considered statistically significant.

## 3 Results and discussions

### 3.1 Synthesis of janus amphiphilic nanoparticles

Janus amphiphilic nanoparticles were successfully synthesized by selectively modifying SiO_2_ nanoparticles at the Pickering emulsion interface, with the exposed side labeled with NH_2_ groups (Fig. 1D). A uniform and stable o/w emulsion was prepared using the Granick’ method (Fig. 1E). Under an optical microscope, the wax droplets appeared uniform, stable, and discrete spherical structures (Fig. 1F). The arrangement of particles around the droplets was analyzed using SEM, which showed that SiO_2_ nanoparticles were evenly distributed on the wax droplet surface, with one side embedded in paraffin (Fig. 1G). Silver nanoparticles were used to selectively label the amino-functionalized regions of SiO_2_ nanoparticles to validate the successful selective modification. The results confirmed unreacted SiO_2_ nanoparticles were not labeled (Fig. 1H). For paraffin-coated SiO_2_ nanoparticles, silver nanoparticles labeled only the exposed NH_2_-modified side, while the paraffin-embedded side remained unlabeled (Fig. 1I). In contrast, fully amino-functionalized SiO_2_ nanoparticles were uniformly labeled with silver nanoparticles on all surfaces (Fig. 1J). These findings demonstrate the successful NH_2_ labeling of the exposed surface of SiO_2_ nanoparticles.

### 3.2 Characterization of janus amphiphilic nanoparticles

**Fig. 2:**
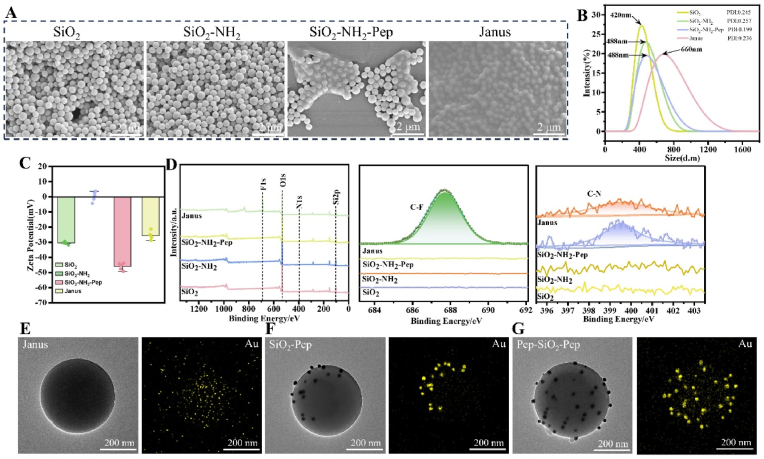
(A) Representative SEM images of different grafting reactions of SiO_2_ Janus particles. (B) Size distribution of the corresponding SiO_2_ particles. (C) Surface potential change of the corresponding SiO_2_ particles. (D) XPS spectra of the corresponding SiO_2_ particles. (E) TEM image of Janus nanoparticles (left), EDS mapping analysis of Au element (right). (F) TEM image of Au nanoparticle-labeled Janus nanoparticles (left), EDS mapping analysis of Au element (right). (G) TEM image of Au nanoparticle-labeled peptide-modified SiO_2_ nanoparticles (left), EDS mapping analysis of Au element (right).

The formation process of amphiphilic SiO_2_ Janus nanoparticles was analyzed using SEM and DLS to evaluate size and morphology changes. Monodisperse silica nanoparticles of specific sizes were successfully synthesized *via* the Stöber method (Fig. 2A). Following surface modification with APTES, the amino-functionalized SiO_2_ nanoparticles retained their uniform morphology. DDDEEKC-Peg(2000)-COOH was grafted onto the nanoparticle surface, forming a uniform film. Upon grafting FOTS to the opposite hemisphere, the DDDEEKC-Peg (2000)-COOH side faced outward, likely due to the autonomous loading behavior of amphiphilic nanoparticles [25,40]. DLS measurements confirmed an increasing size trend during the formation of SiO_2_ Janus particles (Fig. 2B), consistent with SEM observations. As shown in Fig. 2C, the zeta potential of the SiO_2_ nanoparticles underwent distinct changes at each modification step. The potential shifted from −30.532 mV (Si-O-) to 0.244 mV (NH_4_^+^) after amino modification, then to −46.068 mV (COO^−^) after Pep coating, and finally to −25.482 mV following FOTS grafting. These results corroborate successful sequential surface modifications. X-ray photoelectron spectroscopy (XPS) provided additional evidence, analyzing the electronic binding energy and surface chemical composition. The F1s spectrum displayed a binding energy at 687.6 eV (CF_2_), while the N1s spectrum showed a binding energy at 399.9 eV (CN) (Fig. 2D), confirming the presence of the functional groups. These results collectively demonstrate the successful synthesis and precise characterization of amphiphilic SiO_2_ Janus nanoparticles. To verify the success of selective modification, sodium citrate-modified Au nanoparticles were used to selectively label the peptide amino regions of the amphiphilic Janus nanoparticles. The TEM image of Janus nanoparticles reveals their preserved spherical morphology. Notably, the Au elemental EDS mapping failed to demonstrate Au distribution (Fig. 2E). Au nanoparticles were employed to specifically label the polypeptide amino regions of amphiphilic Janus nanoparticles to verify the success of selective modification. The results demonstrate that one hemisphere of the amphiphilic Janus nanoparticles was successfully labeled with Au nanoparticles while the opposite hemisphere remained unmodified. Corresponding Au elemental EDS mapping clearly shows Au distribution exclusively on one side of the nano-silica particles (Fig. 2F). In contrast, DDDEEKC-Peg(2000)-COOH modified SiO_2_ nanoparticles exhibited complete surface labeling by Au nanoparticles, with EDS mapping revealing homogeneous Au distribution across the entire silica surface (Fig. 2G). These findings conclusively demonstrate successful unilateral modification of hydrophilic polypeptide chains on the SiO_2_ nanoparticles.

### 3.3 Physicochemical properties of janus amphiphilic nanoparticles coated enamel slices

**Fig. 3:**
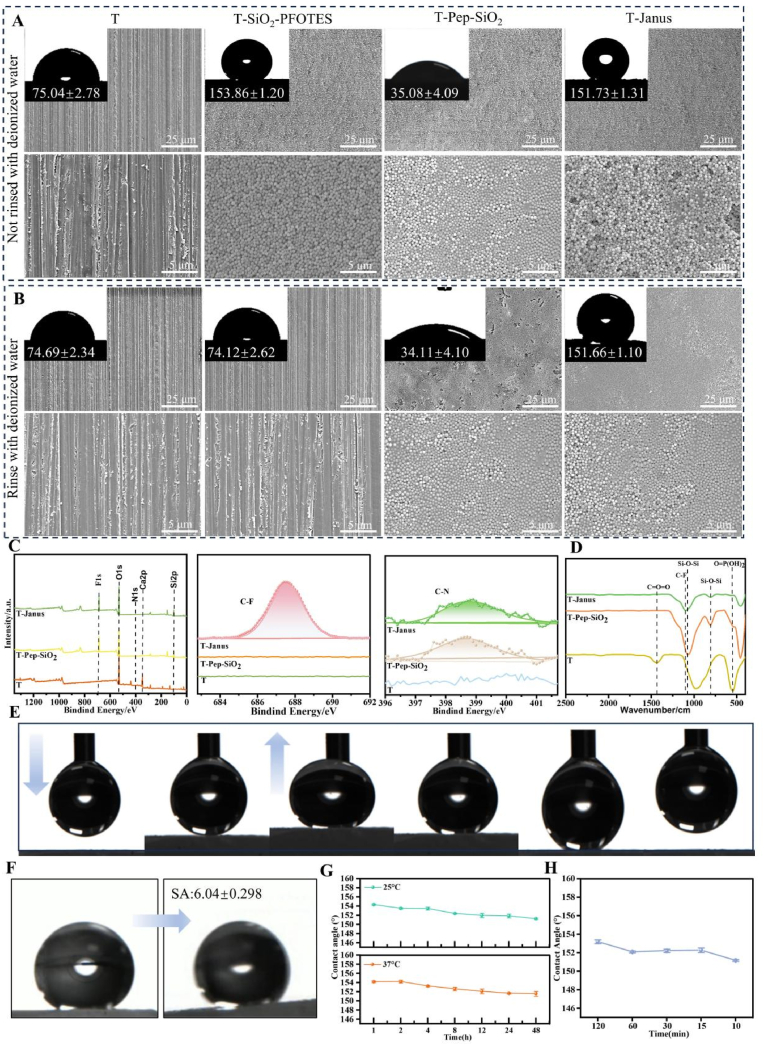
Water contact angles and SEM images of different treated enamel slices of (A) unwashed and (B) washed groups of enamel slices. (C) The XPS image of the rinsed enamel slice. (D) The FTIR spectra of the rinsed enamel slice. (E) The low viscosity between the Janus particle coating and the water droplets. (F) Water rolling angle of T-Janus. (G) Exploration of superhydrophobic stability of Janus particles. (H) Water rolling angle of Janus amphiphilic particles. Formation time of superhydrophobic coating of Janus particles.

The adhesion and hydrophobic properties of Janus amphiphilic nanoparticles on enamel slices were analyzed by measuring water contact angles and observing surface morphology using SEM. The initial hydrophobic angles of unwashed enamel slices, T-SiO_2_-FOTS, T-Pep-SiO_2_, and T-Janus coated enamel slices were 75.04 ± 2.78°, 153.86 ± 1.20°, 35.08 ± 4.09°, and 151.73 ± 1.31°, respectively (Fig. 3A). After rinsing, the hydrophobic angle for T-SiO_2_-FOTS coated enamel decreased to 74.12 ± 2.61° (Fig. 3B). SEM imaging revealed that the SiO_2_ coating on T-SiO_2_-FOTS enamel slices was removed after rinsing, while Janus particle-coated slices retained SiO_2_. These findings confirm that Janus particles exhibit both superhydrophobicity and strong adhesive properties.

Further characterization of adsorption characteristics on enamel slices coated with T-Pep-SiO_2_ and T-Janus particles was conducted using SEM, EDS, XPS, FTIR, and hydrophobicity tests. XPS spectra (Fig. 3C) revealed the characteristic binding energy of CF_2_ at 687.6 eV on enamel slices coated with Janus particles, while both T-Pep-SiO_2_ and T-Janus coated slices displayed a CN binding energy at 398.5 eV. FTIR analysis (Fig. 3D) identified characteristic peaks at 1096 cm^−1^, 800 cm^−1^, and 496 cm^−1^ corresponding to the antisymmetric stretching, symmetric stretching, and bending vibrations of the Si-O-Si group, respectively, confirming the presence of SiO_2_. A peak at 600 cm^−1^ indicated the bending vibration of the P-O bond, signifying the successful anchoring of the DDDEEKC polypeptide on the SiO_2_ surface. After FOTS modification, the 1096 cm^−1^ peak shifted to 1101 cm^−1^, corresponding to the C-F stretching vibration. EDS results showed a decrease in calcium, phosphorus, and carbon and an increase in silicon and oxygen in the T-Pep-SiO_2_ group compared to the uncoated enamel (T group). In the T-Janus group, silicon, oxygen, and fluorine increased compared to T-Pep-SiO_2_, indicating successful FOTS modification (Fig. S1).

To further confirm the superhydrophobic properties of Janus particle-coated enamel slices, rolling angle measurements showed a low rolling angle of 1.95 ± 0.25° between the water droplets and the T-Janus particle surface (Fig. 3E and F, Video ESM1,2). Stability tests demonstrated that T-Janus-coated enamel slices maintained a superhydrophobic water contact angle above 151° for at least 48 h at 25 °C and 37 °C (Fig. 3G), confirming excellent stability and durability.

Supplementary data related to this article can be found online at https://doi.org/10.1016/j.mtbio.2025.101627

The following are the Supplementary data related to this article.

Video 1 2 Video 1

Video 2 3 Video 2

Considering the effect of the salivary-acquired pellicle on Janus particle adhesion, enamel slices (n = 3 per group) were pre-soaked in artificial saliva to simulate *in vivo* conditions [[41], [42], [43]]. These slices were then immersed in a Janus particle solution for varying durations (10, 15, 30, 60, and 120 min). Water contact angles for all slices remained above 151° (Fig. 3H), demonstrating that Janus particles maintained strong adhesion and superhydrophobicity even in the presence of the acquired pellicle. These results confirm that Janus amphiphilic nanoparticles exhibit excellent superhydrophobic properties, strong adhesive capabilities, and durability under simulated oral conditions.

### 3.4 Biofilm inhibition experiment

**Fig. 4:**
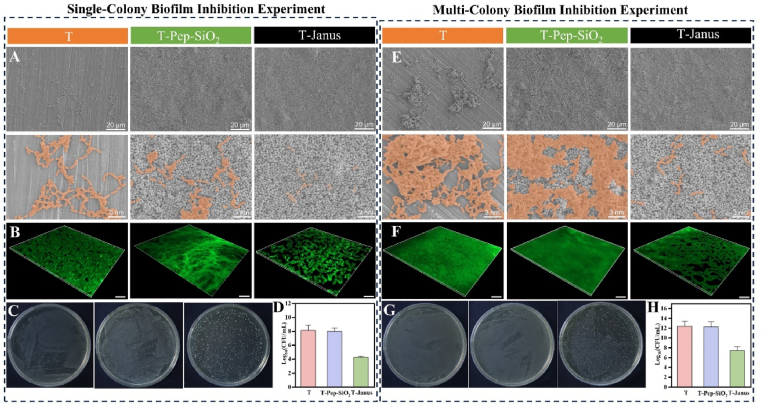
Single colony biofilm inhibition experiment: (A) SEM images after co-cultivation of enamel slices with different surface treatments and *S.mutans*. (B) Three-dimensional CLSM images after co-cultivation (scale: 25 μm). (C) photos of biofilm colonies after co-cultivation (dilution ratio 1:100). (D) corresponding CFU count of biofilm. (E) SEM images after co-cultivation enamel slices with different surface treatments and *S. sobrinus, S. sanguinis, and S. mutans.* (F) Three-dimensional CLSM images after co-culturing(scale: 25 μm). (G) photos of biofilm colonies after co-culture (dilution ratio 1:10000). (H) corresponding CFU counts of biofilm.

Both single-colony and multi-colony biofilm experiments were conducted to evaluate the biofilm-inhibitory effects of Janus amphiphilic nanoparticles. Although plaque biofilms are not fully mature after 8 h of cultivation, bacteria can form membrane-like structures and synthesize related substances on growth substrates [8,44,45]. SEM, laser confocal microscopy, bacterial coating, and CFU counting were used to analyze the growth of Streptococcus mutans on enamel plates co-cultured with T, T-Pep-SiO_2_, and T-Janus groups for 8 h. As shown in Fig. 4A, the T-Janus group exhibited the lowest adhesion of *S. mutans*. CLSM imaging (Fig. 4B) revealed that *S. mutans* adhered difficultly to the T-Janus-coated enamel surface. Additionally, colony count results confirmed the significant reduction in *S. mutans* biofilm formation on T-Janus coated enamel (Fig. 4C and D). Results from the multi-colony biofilm inhibition experiments were consistent with the single-colony experiments, confirming that Janus nanoparticle coatings effectively inhibit the adhesion of streptococci to enamel.

### 3.5 Mineralization performance *in vitro*

**Fig. 5:**
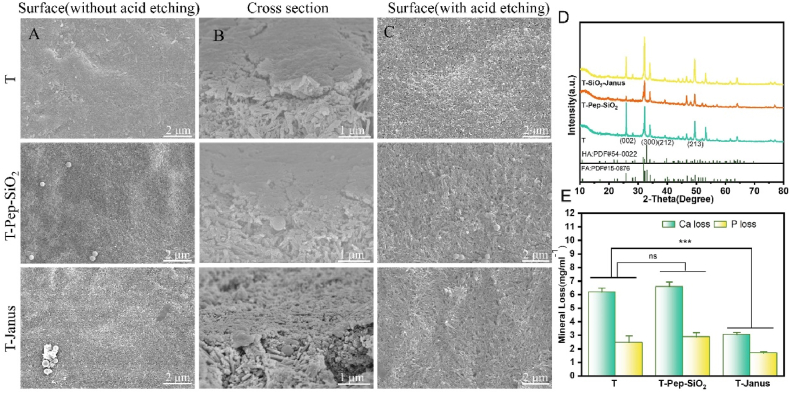
(A) SEM images of the surface of T, T-Pep-SiO_2_, and T-Janus coated enamel slices after 5 days of remineralization. (B) SEM images of the longitudinal section of enamel after 3 days of remineralization. (C) SEM images of remineralized enamel after etching with 6 wt% citric acid for 1 min. (D) XRD pattern of enamel after 3 days of remineralization. (E) Concentration loss of calcium and phosphorus ion in remineralized enamel following citric acid etching. (ns, *p* > 0.05; ∗∗∗, *p* < 0.001).

The mineralization properties of Janus nanoparticle coatings on enamel were analyzed using SEM, XRD, and ICP-OES. SEM images (Fig. 5A) showed that mineralized crystals selectively grew on the enamel surface, covering honeycomb-like micropores formed by demineralization and creating an enamel-like structure. No significant differences in the mineralized enamel interfaces were observed among the T, T-Pep-SiO_2_, and T-Janus groups, likely due to fluoride ions promoting mineralization at the hydrophobic interface [6,46,47]. Longitudinal SEM sections (Fig. 5B) revealed a mineralized layer on the enamel surface of all three groups, with clear boundaries between the mineralized layer and the underlying enamel. The crystals were small, densely packed, and disordered. XRD analysis (Fig. 5D) identified diffraction peaks in the remineralized material at 2θ of 25.9° (002), 32.9° (300), 39.9° (212), and 49.5° (213), corresponding to HA (PDF #54-0022) and fluorapatite (FA; PDF #15–0876). Peaks in the T-Janus group at 2θ of 25.9–26.1° and 31.9–33.0° were highly concentrated and sharp, indicating higher HA and FA crystallinity compared to other groups. In the acid resistance test (Fig. 5C), SEM images revealed significant demineralization and micro-void formation on the enamel slices of all groups, with crystals partially dissolved, likely due to incomplete mineralization [17,41,48]. ICP-OES analysis (Fig. 5E) showed that calcium and phosphorus losses in the acid-etched T-Janus coated enamel slices were significantly lower than those in uncoated enamel slices (Ca: 3.038 ± 0.174 vs. 6.182 ± 0.302; P: 1.680 ± 0.109 vs. 2.457 ± 0.493; *p* < 0.05). There were no statistical differences in calcium and phosphorus losses between T and T-Pep-SiO_2_ groups (*p* > 0.05). These results demonstrate that remineralized minerals in the T-Janus group provide enhanced protection against acid erosion and can shield deeper tooth tissues from damage.

### 3.6 Biocompatibility assessment

**Fig. 6:**
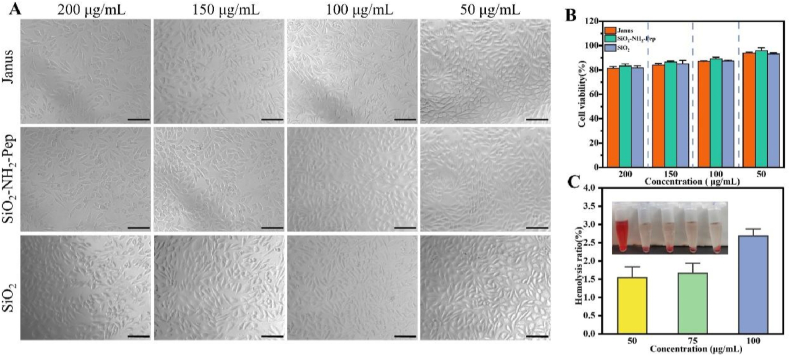
(A) *In vitro* safety evaluation. Cell morphology (scale: 100 μm) and (B) cell viability of CCK-8 after treatments with different doses of SiO_2_, SiO_2_-Pep, and Janus nanoparticles for 1 day, respectively. (C) The hemolysis rate of Janus nanoparticles at different concentrations.

Prior to their application in biomedicine, the toxicity of nanomaterials must be rigorously evaluated. Cytotoxicity was assessed through morphological observation and CCK-8 cell viability assays using L929 fibroblasts treated with varying concentrations of SiO_2_, SiO_2_-Pep, and Janus nanoparticles. As shown in Fig. 6A, the cells grew in tight rows with no significant extension of filamentous structures, indicating normal morphology. Cell viability in all groups remained above 80 %, as measured by CCK-8 (Fig. 6B), demonstrating the intrinsic cellular compatibility of the superhydrophobic Janus nanoparticles.

Given the potential for gingival bleeding due to inflammation, the blood compatibility of anti-caries materials was evaluated. Hemolysis ratios for all groups were below 5 % (Fig. 6C), meeting the threshold for good hemocompatibility. In contrast, the positive control (pure water) showed red discoloration due to red blood cell lysis caused by osmotic pressure. These results confirm that Janus nanoparticles possess excellent blood compatibility, making them suitable for biomedical applications. In addition, we further validated its biosafety using a co-culture method, and the results remained consistent (Fig. S2).

### *3.7* *In vivo* application and biocompatibility assessment

To evaluate the anti-caries effect of self-adhering superhydrophobic Janus particles *in vivo*, 6-week-old rats were used in the experiment. Exogenous flora was introduced into the oral environment, and the rats were treated with Janus particles. The occlusal surfaces of teeth were observed, and the extent and severity of dental caries were assessed using the Keyes scoring method.

**Fig. 7:**
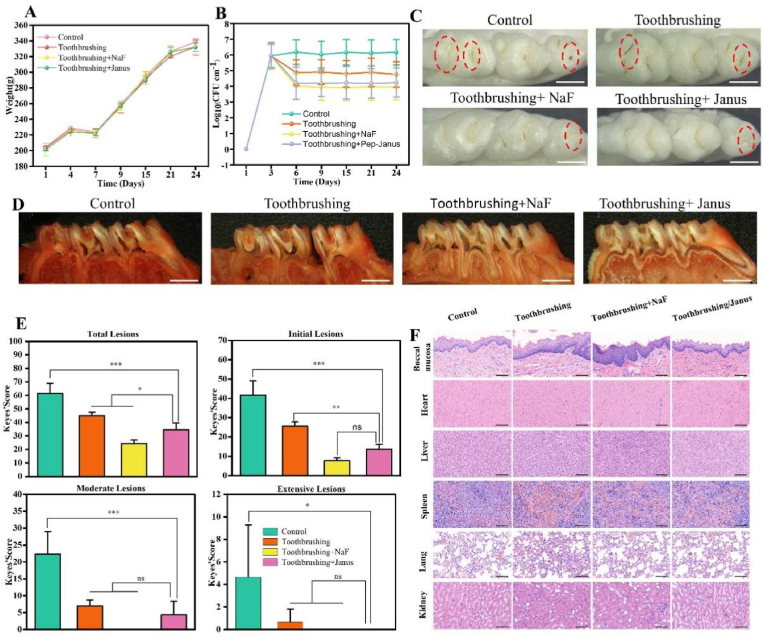
Evaluation of the anti-caries effect of Janus *in vivo*. (A) The body weight changes of rats in each group. (B) The CFU counts of each group. (C) The occlusal surface images of teeth in each group on the 24th day (Ammonium violurate staining images, 20 μm). (D) Various groups of dental caries models. (Ammonium violurate staining images, 20 μm). (E) Statistical results of the degree and depth of caries at the pit and fissure points and sutures of each group (ns, *p* > 0.05; ∗, *p* < 0.05; ∗∗, *p* < 0.01; *∗∗∗*, *p* < 0.001). (F) H&E staining results of buccal mucosa and main organs (scale: 100 μm).

Body weight measurements indicated no significant differences among the groups, with a steady weight increase throughout the experiment, suggesting the *in vivo* biological safety of the Janus particles. The transient weight loss observed on the third day may reflect the Rat adapting to a new diet, as confirmed by subsequent weight recovery and growth (Fig. 7A). Bacterial CFU counts in oral samples (Fig. 7B) revealed that exogenous bacteria successfully colonized the oral cavity, establishing the infection model. The Janus particle-treated group exhibited significantly greater bacterial inhibition than the brushing group, reducing bacterial levels by approximately 0.7 orders of magnitude. The antibacterial effect in the blank control group was weak, even less effective than the sodium fluoride group. Occlusal surface images of dental caries (Fig. 7C) showed visible pulp cavities in the third molars of the blank control group and enamel caries in the brushing group. Application of Janus particles after brushing reduced caries severity, demonstrating a clear improvement compared to the control and brushing-only groups.

To further assess caries severity, samples were stained with 0.4 % ammonium violet urea (Fig. 7D), and the lesions were scored using the Keyes scoring system. The scores categorized caries into four levels: total lesions (E + Ds + Dm + De), initial lesions (Ds + Dm + De), moderate lesions (Dm + De), and severe lesions (De). As shown in Fig. 7E, the Pep/APTES Janus particle group exhibited significantly reduced caries severity, with lesions primarily confined to the enamel layer and no severe lesions observed.

Histological evaluation of major organs using H&E staining showed no abnormalities after 24 days of treatment (Fig. 7F), further confirming the biocompatibility of Janus particles. These results collectively demonstrate that Janus particles possess notable anti-caries properties while maintaining excellent *in vivo* biocompatibility.

The static water contact angles of rat molars at different time points were measured to simulate the impact of the oral environment on the coating's retention time (Fig. S3). The water contact angle for molars without amphiphilic Janus nanoparticle coating was 79.23 ± 0.88°, while that for molars coated with amphiphilic Janus nanoparticles was 158.51 ± 1.27°. After 2 and 4 h of retention in the oral cavity, the water contact angles of the coated molars decreased to 155.42 ± 1.23° and 152.06 ± 0.67°, respectively. Although the amphiphilic Janus nanoparticles retained their superhydrophobic properties after 4 h of intraoral retention, prolonged exposure gradually reduced water contact angles on tooth surfaces. This observation suggests that normal masticatory movements and salivary flushing in the oral environment may compromise the coating's stability.

## 4 Conclusions

This study successfully designed and synthesized Janus amphipathic particles with both enamel-specific adhesion and superhydrophobicity. One end of the particles was functionalized with tridecafluorooctyltriethoxysilane to impart superhydrophobicity, while the other end was grafted with DDDEEK-Peg-COOH for specific adhesion to tooth surfaces. However, the specific binding site between the amino groups on SiO_2_ and the carboxyl groups on DDDEEK-Peg-COOH is worthy of investigation. These particles can form a superhydrophobic coating on the enamel surface, reducing the colonization of cariogenic bacteria on the tooth surface and preventing caries. Importantly, they do so without interfering with the remineralization of demineralized enamel.

Furthermore, the biocompatibility assessments revealed no significant cytotoxic effects on cells or adverse impacts on major organs. Compared to traditional antibiotics, Janus particles reduce bacterial resistance. These findings suggest that Janus superhydrophobic particles hold significant potential as a novel, effective, and safe approach for preventing dental caries. Future research could further focus on loading intelligent, responsive antibacterial agents into mesoporous silica to enhance these materials' biological safety and antibacterial properties, maintain oral microecological balance, and mitigate bacterial resistance.

## CRediT authorship contribution statement

**Chenhui Chen:** Writing – original draft, Validation, Methodology, Investigation, Formal analysis. **Yuqi Xie:** Writing – original draft, Methodology, Investigation. **Qinfeiyang Zhang:** Investigation, Data curation. **Junyan Cao:** Methodology. **Jixing Lin:** Investigation. **Xian Tong:** Investigation. **Yihao Wu:** Visualization. **Li Zhu:** Writing – review & editing, Supervision, Conceptualization. **Peng Gao:** Writing – review & editing, Writing – original draft, Supervision, Conceptualization. **Jianfeng Ma:** Writing – review & editing, Supervision, Resources, Funding acquisition.

## Declaration of competing interest

The authors declare that they have no known competing financial interests or personal relationships that could have appeared to influence the work reported in this paper.

## Data Availability

No data was used for the research described in the article.
