# Supplementary material for: Amphiphilic Janus nanoparticles for a superhydrophobic coating on the enamel surface to prevent caries
Source: Mater Today Bio. 2025 Mar 6;32:101627. doi: 10.1016/j.mtbio.2025.101627 (PMC11953956; doi:10.1016/j.mtbio.2025.101627)
Supplement: Multimedia component 1 [file mmc1.docx]

**
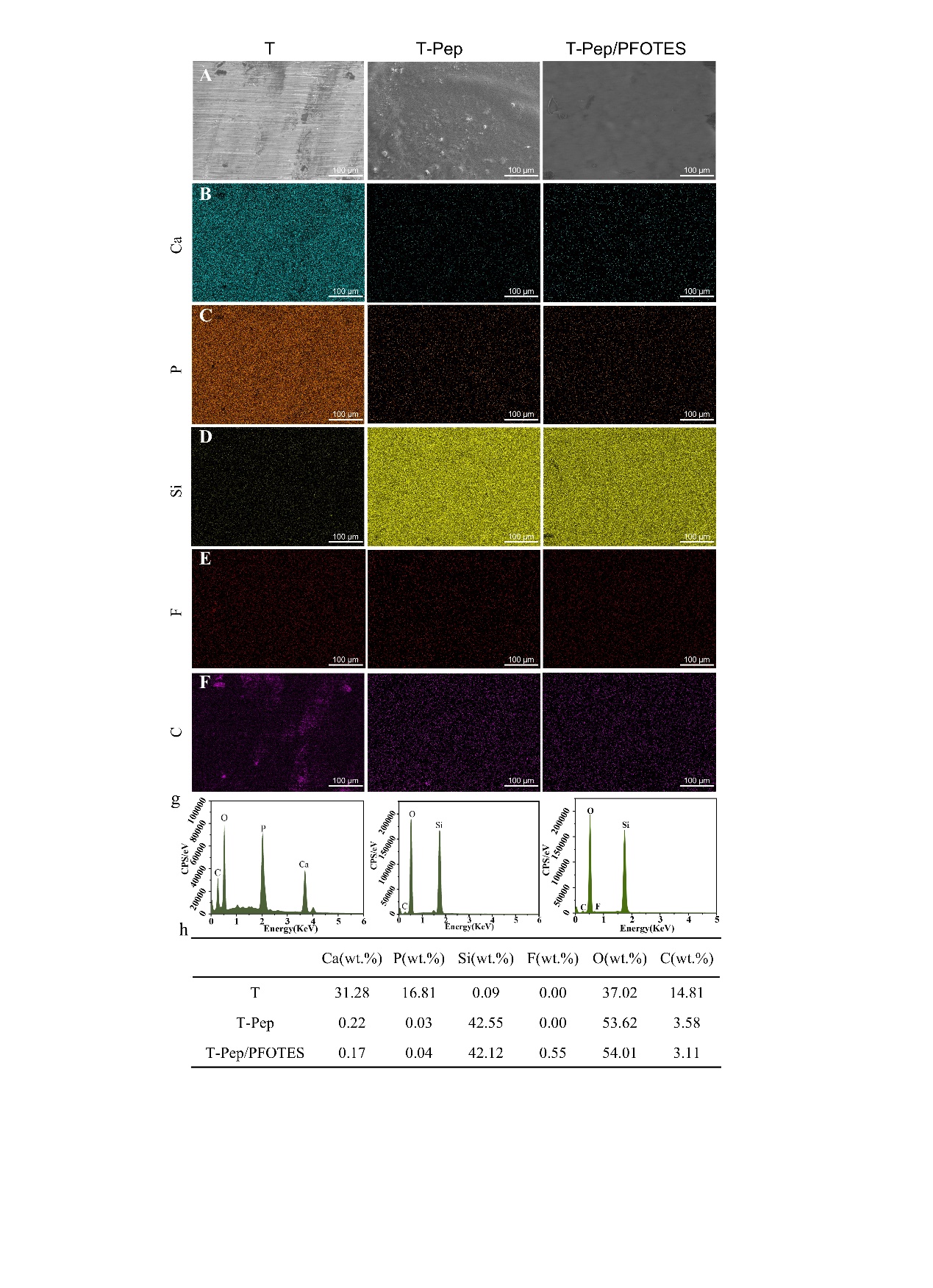
**

**S 1.** EDS images of different enamel slices after washing.


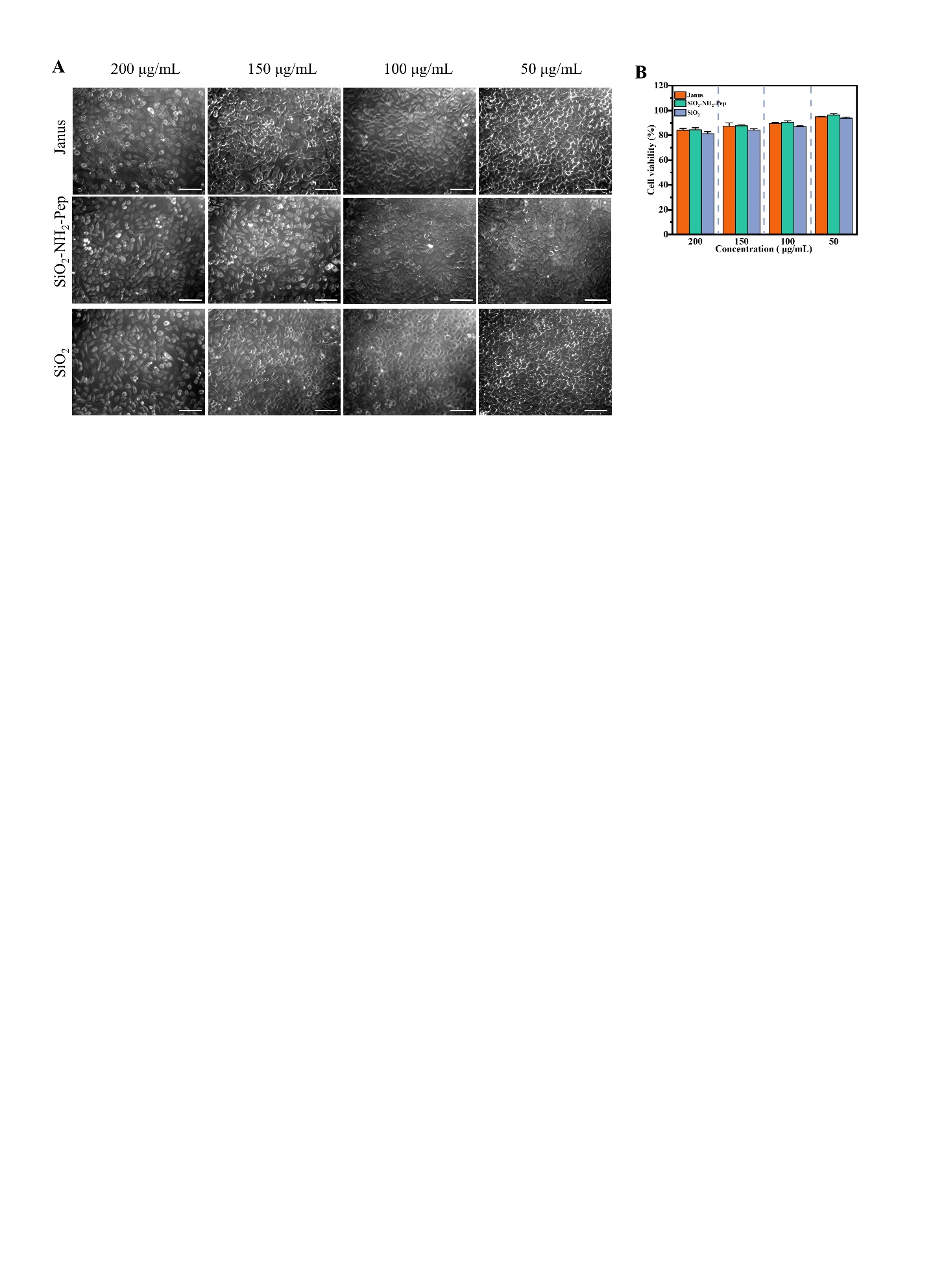


Fig.S2 (A) In vitro safety evaluation. Cell morphology (scale: 100μm), and (B) cell viability of CCK-8 after treatments with different doses of SiO2, SiO2-Pep, and Janus nanoparticles respectively for 24 hours.


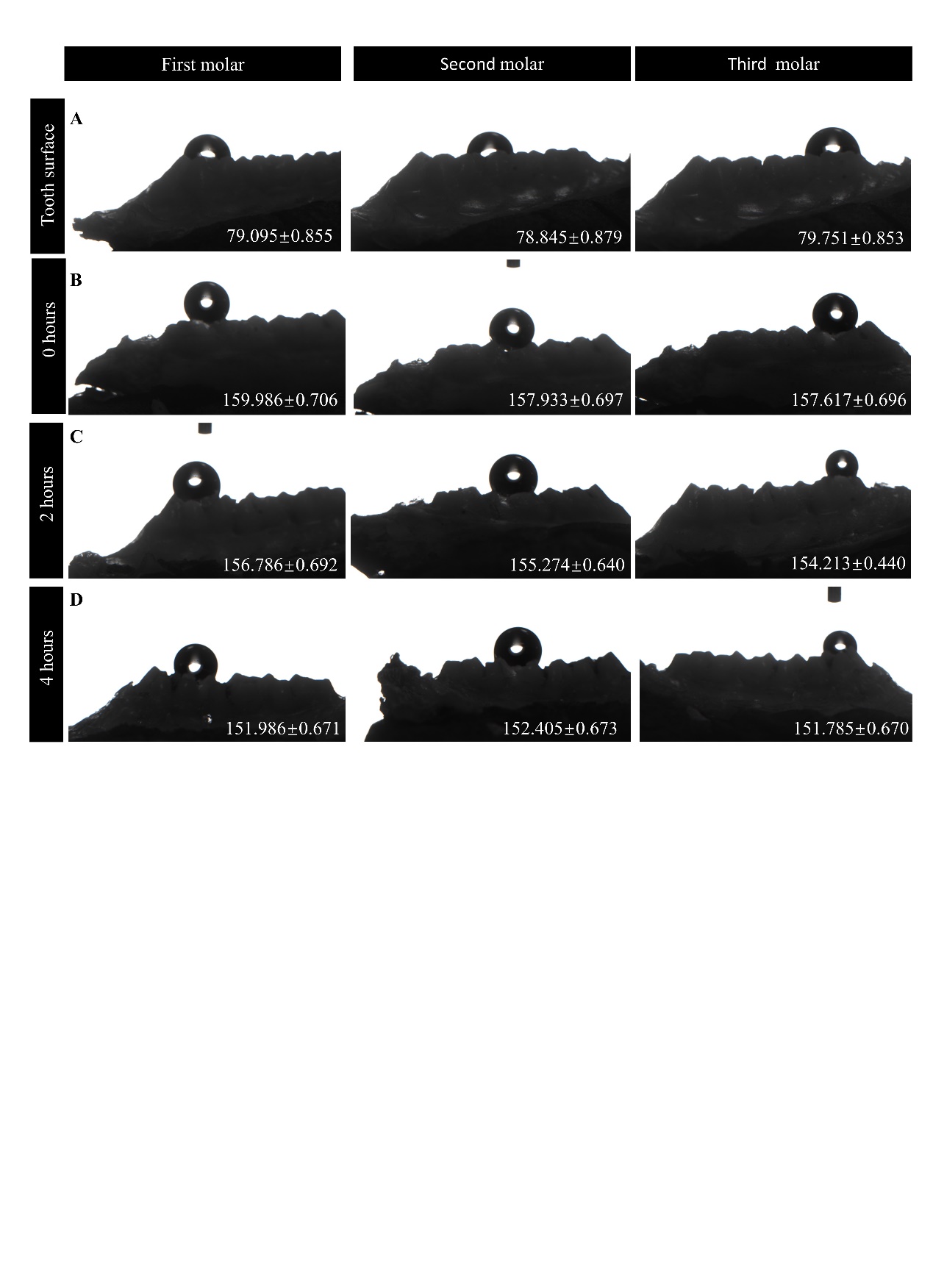


Fig. S3 (A) Water contact angle on the surface of rat molars without the superhydrophobic coating; (B) Water contact angle on the surface of rat molars coated with the superhydrophobic coating; (C) Water contact angle on the surface of rat molars after 2 hours of retention in the oral cavity; (D) Water contact angle on the surface of rat molars after 4 hours of retention in the oral cavity.
